# Correction: Non-guided, mobile, CBT-I-based sleep intervention in War-torn Ukraine: A feasibility study

**DOI:** 10.1371/journal.pone.0337725

**Published:** 2025-11-25

**Authors:** Anton Kurapov, Jens Blechert, Alexandra Hinterberger, Pavlos Topalidis, Manuel Schabus

The Funding statement for this article is incorrect. The correct Funding statement is as follows: This research was fully funded by MSCA4Ukraine and the European Union (Grant to AK), and partially funded by the Austrian Science Fund (FWF; Grant No. 10.55776/W1233 to MS).

## References

[1] Kurapov A, Blechert J, Hinterberger A, Topalidis P, Schabus M. Non-guided, mobile, CBT-I-based sleep intervention in War-torn Ukraine: A feasibility study. PLoS One. 2025;20(5):e0310070. doi: 10.1371/journal.pone.0310070 40424239 PMC12111256

