# Supplementary material for: Non-guided, mobile, CBT-I-based sleep intervention in War-torn Ukraine: A feasibility study
Source: PLoS One. 2025 May 27;20(5):e0310070. doi: 10.1371/journal.pone.0310070 (PMC12111256; doi:10.1371/journal.pone.0310070)
Supplement: Supplementary Tables 1 to 3 — This table lists the questions used to evaluate participants’ acceptance of the program, including perceived effectiveness, clarity of content, usefulness of exercises, and likelihood of recommending the program. (DOCX) [file pone.0310070.s001.docx]

**S1 Table. Formulations of Items Regarding the Acceptance of the Program.** This table lists the 12 custom-designed questions used to evaluate participants' acceptance of the Sleep2Ukraine program, focusing on sleep improvement, program usability, and feature usefulness, as well as participants’ overall satisfaction and willingness to recommend the program.

| **No.** | **Question** |
| --- | --- |
| 1 | Has the quality of your sleep improved after following the proposed program? |
| 2 | How effective was the proposed program for you personally? |
| 3 | Was the content of the application generally clear to you? |
| 4 | Did the content of the exercises address the problems they were supposed to solve? |
| 5 | Did the "sleep quality index" calculated in the application match your subjective feelings about the quality of your sleep? |
| 6 | How accurate do you consider the program’s level distribution? |
| 7 | Was it easy for you to meet all the level requirements to move to the next one? |
| 8 | Were the relaxation exercises useful for you? |
| 9 | Were the visualization exercises useful for you? |
| 10 | Was the chatbot in the application useful for you? |
| 11 | Were the push notifications (background reminders) useful for you? |
| 12 | Would you recommend ‘Sleep^2^Ukraine’ to your family, friends, and acquaintances? |

**S2 Table. Scoring Ranges, Number of Items, Reliability Statistics, and Clinical Cutoff Scores for Psychological Questionnaire Measures.** This table shows names of the variables, corresponding measures, reliability statistics, scoring range, and the number of items per measure.

| **Variable Group** | **Variable** | **Measure** | **Cutoff Score** | **Reliability (Cronbach’s Alpha)** | | **Range (min-max)** | **Number of Items** |
| --- | --- | --- | --- | --- | --- | --- | --- |
|  |  |  |  | T1 | T2 |  |  |
| Sleep | Sleep Disturbance | PSQI | >5 | .72 | .74 | 0-21 | 19 |
|  | Insomnia | ISI | >7 | .84 | .83 | 0-28 | 7 |
|  | Fear of Sleep | FoSI | >15 | .84 | .86 | 0-65 | 13 |
| Mental Health | Anxiety | GAD-7 | >5 | .88 | .89 | 0-21 | 7 |
|  | Depression | PHQ-9 | >5 | .84 | .83 | 0-27 | 9 |
|  | PTSD | PCL-5 | >31 | .93 | .94 | 0-80 | 20 |
|  | Perceived Stress | PSS-4 | >7 | .93 | .94 | 0-16 | 4 |
|  | Somatic Symptoms | SSS-8 | >4 | .74 | .74 | 0-32 | 8 |
|  | Resilience | BRS-6 | <13 | .72 | .68 | 6-30 | 6 |

**S1 Figure. Density Differences between Pre- and Post-Measurements for Program Completers (N = 160).** The red vertical line in the figure indicates clinical cutoff for each measure: non-clinical (left side) vs. clinical (right side) values. Corresponding pre-post change percentages are presented on the left side for non-clinical and on the right side for clinical: percentages represent number of people in every category pre- and post-training. Results are shown for subjective measures for the program completers (N = 160).


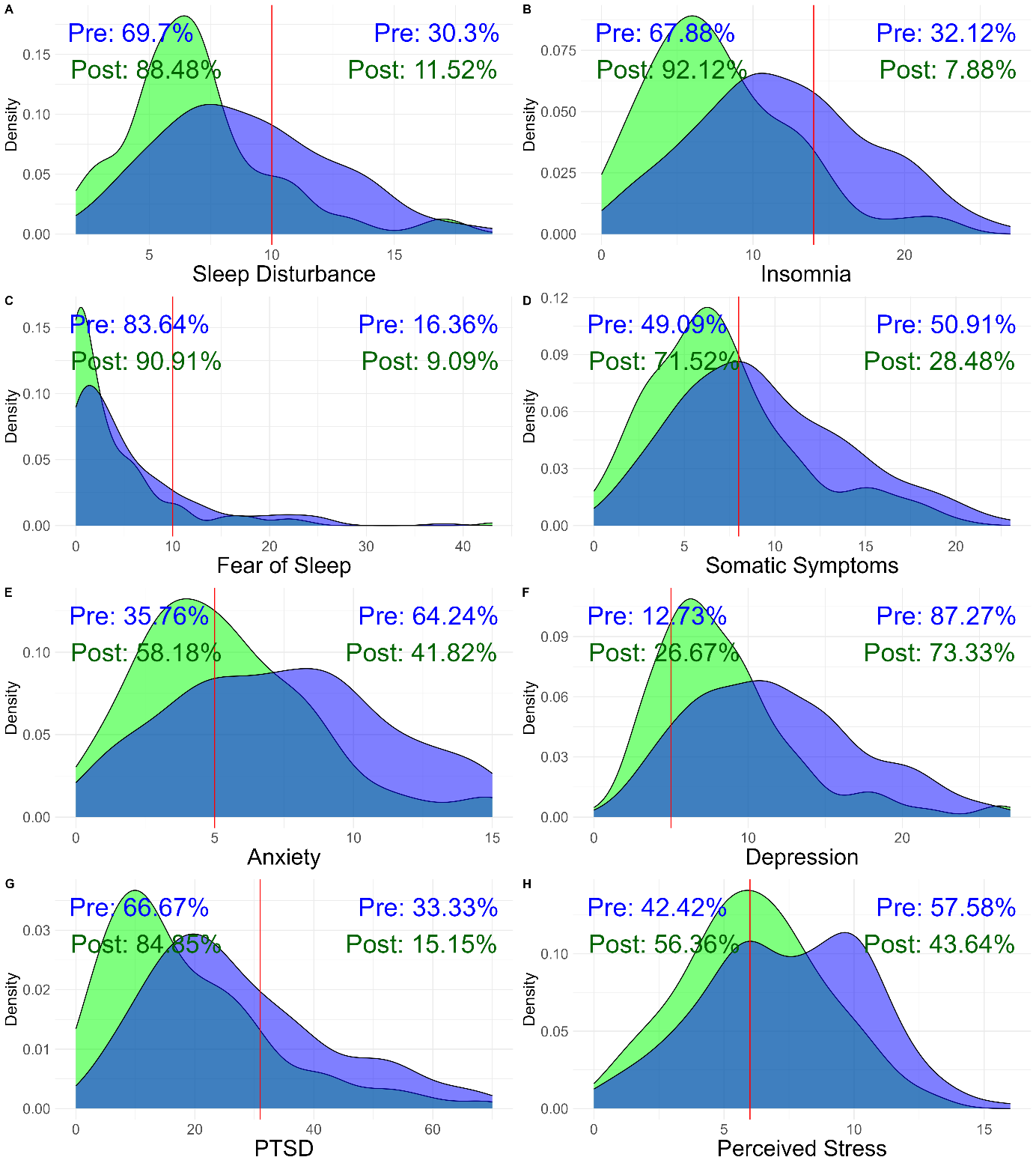

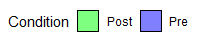


**S3 Table. Changes in Symptoms after the Sleep Coaching for All Participants***.* The table presents the results of paired-sample t-tests for subjective measures obtained with the questionnaires at T0 and T2 (see Figure 1 for details) for all participants (N = 283), including dropouts (N = 123). For dropouts, pre-assessment values were carried forward to the post-assessment, assuming no change due to the intervention, as part of an intention-to-treat analysis in this non-controlled pilot study.

|  | **T0** | **T2** | **T2-T0** | **t(281)** | ***p*** | **Cohen’s *d*** |
| --- | --- | --- | --- | --- | --- | --- |
|  | **(M ± SD)** | **M ± SD)** |  |  |  |  |
| Sleep Disturbance | 8.87 ± 3.58 | 7.67 ± 3.34 | -1.2 | 5.7 | <.001 | .34 |
| Insomnia | 11.98 ± 5.69 | 9.40 ± 5.64 | -2.58 | 6.72 | <.001 | .41 |
| Fear of Sleep | 5.55 ± 6.75 | 4.12 ± 6.26 | -1.43 | 3.59 | <.001 | .21 |
| Anxiety | 7.18 ± 3.71 | 6.03 ± 3.44 | -1.15 | 4.9 | <.001 | .29 |
| Depression | 11.76 ± 5.55 | 9.74 ± 5.30 | -2.02 | 5.63 | <.001 | .34 |
| PTSD | 27.08 ± 15.42 | 21.73 ± 15.06 | -5.35 | 5.64 | <.001 | .34 |
| Stress | 7.46 ± 2.97 | 6.58 ± 2.80 | -0.88 | 4.56 | <.001 | .27 |
| Somatic Symptoms | 9.29 ± 4.62 | 7.73 ± 4.16 | -1.56 | 5.1 | <.001 | .31 |
