# Supplementary figures and images for: Non-guided, mobile, CBT-I-based sleep intervention in War-torn Ukraine: A feasibility study
Source: PLoS One. 2025 May 27;20(5):e0310070. doi: 10.1371/journal.pone.0310070 (PMC12111256; doi:10.1371/journal.pone.0310070)

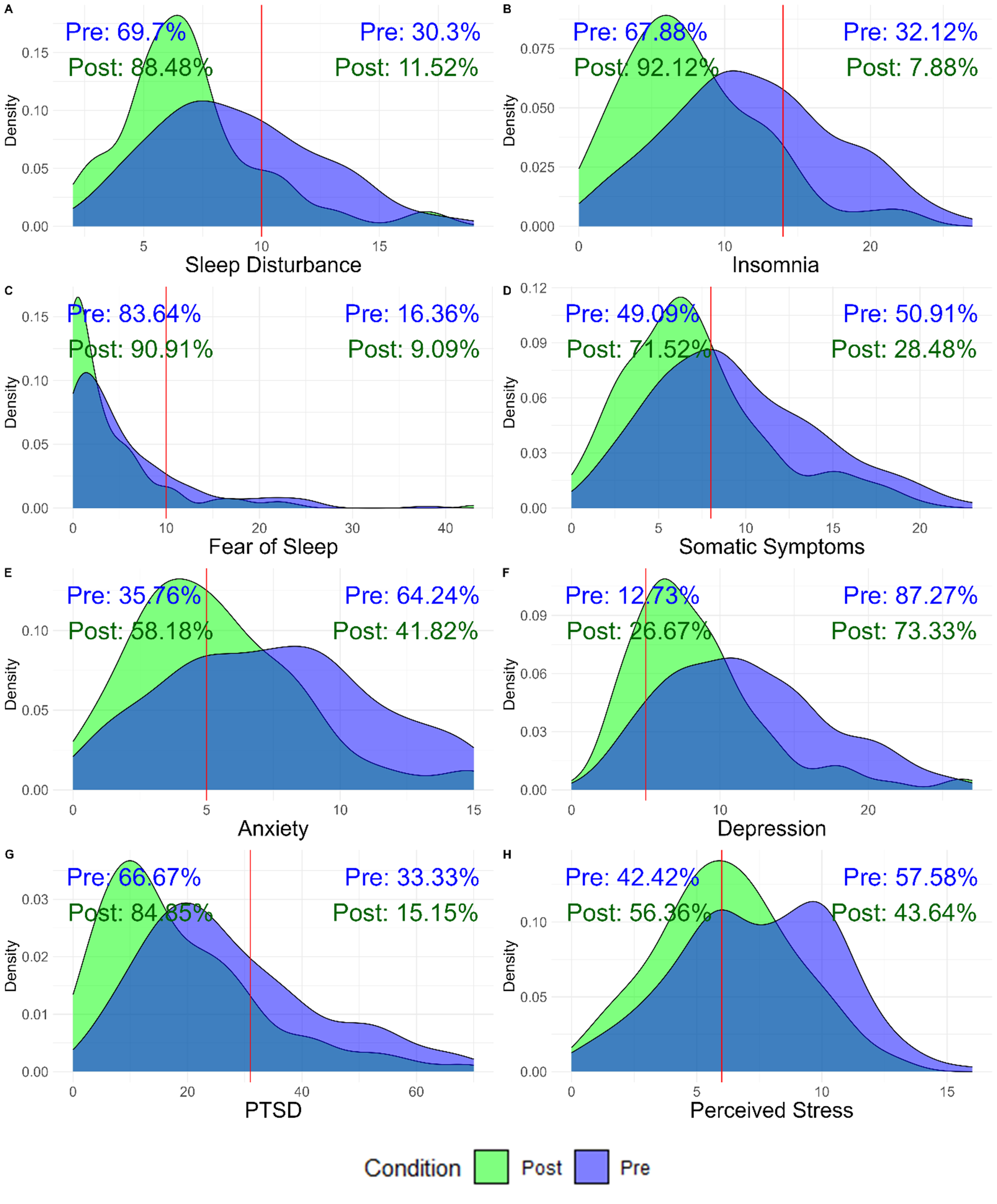

Supplement: Supplementary Fig 1 — This figure illustrates the density differences in subjective measure scores between pre- and post-program assessments for participants who completed the program. The red vertical line indicates the clinical cutoff. Percentages of participants in clinical and non-clinical categories pre- and post-intervention are shown. (TIF) [file pone.0310070.s002.tif]
