# Supplementary material for: Non-guided, mobile, CBT-I-based sleep intervention in War-torn Ukraine: A feasibility study
Source: PLoS One. 2025 May 27;20(5):e0310070. doi: 10.1371/journal.pone.0310070 (PMC12111256; doi:10.1371/journal.pone.0310070)
Supplement: Attachment — Submitted filename: Response to Reviewers.docx [file pone.0310070.s004.docx]

**Response to Reviewers:**

Dear Reviewer 1,

We highly appreciate your detailed review, please find our responses below:

1.1. Full and short title is the same. It is supposed to be different.

*Response*: thank you, adjusted.

2.2. In the abstract the background section lacks, and it is not really understandable. The authors should rewrite it in a more linear way.

*Response*: Abstract has been revised to provide more details and make the description more linear.

Introduction:

2.3. I suggest removing the subheadings.

*Response*: thank you, subheadings have been removed.

2.4. Line 98-99: please specify the sleep parameters and mental health indices.

*Response*: thank you, this has been elaborated in the text.

2.5. Line 138: the first paragraph of method section should be the participants section, in which it should be described participants inclusion and exclusion criteria and sample characteristics.

*Response*: thank you for pointing it out, corresponding section with all necessary details has been added.

2.6. Line 141: specify which are the t0, t1 and t2 measures and how long does it take each t. Furthermore, even if the study plan is described in Figure 1, I suggest describing the study design more deeply in the text. For example, a brief description- even in bullet points- of the time intervals (t0, t1 and t2) and the relative measures acquired at each point.

*Response:* thank you for mentioning, some little details have been added in the “Study design” and “Measures” section, even though we attempted to keep the repetition at low.

2.7. Line 191 and 196: I suggest explaining deeply the questionnaire and to do it in bullet point, instead of bold. It could be more easily to the reader to understand. Furthermore, the PSQI does not measure sleep disturbances but subjectively perceived sleep quality. Did the authors control for cutoff scores? On what bases sample is divided in sleep disturbances and chronic sleep problems? Authors should better describe the sample data regarding sleep and health intext, describing score obtained at t0 and at t2, if reached scores under the cutoff levels.

*Response:* thank you for pointing out these details, corresponding adjustments have been made, hope the sections reads better and clearer now. Obtained scores are reported in results section.

2.8. For health data (table 2) no participants have scores under the cutoff?

*Response:* thank you for pointing it, indeed seems necessary to add, corresponding change has been made.

2.9. Line 205: explain how the HR works and how it works in relation to sleep.

*Response:* thank you, adjusted in the methods section, subsection “Objective sleep measurement”

2.10. Line 215: explain/describe daily/night data.

*Response*: thank you, corresponding adjustments have been made in methods section, hope should be clearer now*.*

2.11. Line 279: specify in the text if the improvement of symptoms reached to clinical values.

*Response:* thank you, correspidning statement has been added.

2.12. Line 292: specify if SOL reduction is statistically significant, and the other measures?

*Response:* thank you, other measures are not, corresponding adjustments have been made in the text.

2.13. The authors should specify and describe in the text the CBT-I components comprised in the app.

*Response:* they have been briefly described + we refer to the table in the supplements that provides more detailed overview. It has been moved there in order not to grab too much attention ot the program itself since this is not the main goal of the current manuscript.

Dear Reviewer 2,

We highly value your very detailed response and suggestions that have been taken into consideration. Please find our responses below:

Abstract

2.1. It would be nice to state in the abstract that there were 160 completers and to specify the proportion that this number represents on the total number of starters.

*Response*: Thank you, added.

2.2. I suggest making it clearer that the objective measure of sleep was provided by the ambulatory heart rate sensor, as this is one innovation of this study.

*Response*: thank you, added several measures here

2.3. As I will detail later, I disagree with the conclusion that effect sizes for subjective measures were all in the medium to high range. I suggest revising this and adding the actual range of effect sizes obtained.

*Response*: thank you, adjusted accordingly.

2.4. I don’t agree with the conclusion that “These findings demonstrate the potential of scalable mobile-based CBT-I…with or without the instrument, based on the heart rate assessment”. This aspect of the intervention was among the least appreciated by the study participants. I would rephrase this to take that finding into account.

*Response*: thank you, conclusion has been rephrased, removing the emphasis form the ‘instrument’.

Introduction

2.5. What is unclear, and could be clarified, is if this study is the first to assess the efficacy of CBT-I, regardless of the treatment format, in people facing war.

*Response*: adjusted in the last paragraph of the introduction.

2.6. What does “low threshold” mean when describing the advantages of a mobile-based CBT-I?

*Response*: clarified in the text.

2.7. Given that heart rate is described as able to infer sleep stages, I find it surprising that sleep stages data were not reported in this manuscript.

*Response*: thank you for pointing out this important aspect: sleep staging was not reported here due to the complexity of the analysis and inability to generalize the data in a proper manner; thus, it has been postponed until to another manuscript. Corresponding adjustments have been made in the “Measures” section.

2.8. High uptake, feasibility and acceptance were hypothesized but are not operationalized. Were criteria to conclude about “high levels” determined a priori, before the analysis?

*Response*: uptake and feasibility are the terms that are generally used in similar pilot studies, thus, require no operationalizations. Corresponding adjustments in the text have been made.

Methods

2.9. There is no mention of the recruitment method used. In addition, what were the inclusion and exclusion criteria? These are not mentioned. This is important in order to know to what extent results may be generalized to the whole population.

*Response*: thank you for pointing it out, corresponding section with all necessary details has been added.

2.10. Also, it seems that the presence of clinically meaningful sleep problems was not required for participation. This needs to be taken into account when interpreting the data. One may not expect the same magnitude of effect sizes if a significant proportion of participants were non-clinical cases at baseline.

*Response*: indeed, it has been brought up in the discussion section.

2.11. Was the content of CBT-I adapted to this population or was it a generic CBT-I?

*Response*: thank you for clarifying, the content remained generic, adaptations mainly concerned formulations. We did not bring many details about adaptation here since were willing to create a separate manuscript for it, but nevertheless some details have been added to the current text.

2.12. It is unclear what acceptance tool the authors adapted to assess acceptance in this study.

*Response:* this point has been slightly elaborated in “Methods” section.

2.13. Several criteria are stated to identify completers (e.g., completed five audio exercises, etc.). However, what proportion of the total content do these represent?

*Response:* thank you for bringing it up, content is equally distributed among all 6 levels (e.g., 2 different relaxation exercises per module, 1 imaginary exercise, 1 chatbot, 1 psychoeducational video, 2 blog articles). Considering different modalities of the content, it is not really feasible to specify the percentage from the total content, but criteria for completion have been elaborated in the text.

2.14. Could the authors explain why they chose an anonymous collection of data?

*Response:* elaborated in methods section.

2.15. One weakness is the translation of the measurement tools by the authors. A more systematic translation approach would have been needed.

*Response:* thank you for mentioning, indeed important aspect, but we have conducted a standardized forward-backward translation (also elaborated better in the manuscript), all corresponding quality measures have been assessed, so the results, obtained by the measures, could have been analyzed and presented.

2.16. Could the authors explain briefly Topalidis et al.’s method to analyse the HR data? They refer to another publication but, as this method is not well known, I suggest explaining it briefly.

*Response:* thank you for mentioning, Elaborated in the text*.*

2.17. For each measure, the authors need to state what cut-off scores they used to determine the presence of clinical levels of symptoms.

*Response:* thank you for specifying, adjusted accordingly in the text and in supplemental tables.

2.18. Please specify how the qualitative feedback was collected and among how many participants.

*Response:* added to the corresponding section, thank you.

Results

2.19. Rather than saying, “there were no obvious differences…”, please rephrase as follows: “there were no significant differences…” (p. 9, 3rd paragraph).

*Response:* adjusted, thank you.

2.20. What was the variable “displacement status” and how was that operationalized?

*Response:* displacement status refers to internally displaced persons (IDP), it has been slightly adjusted in methods section to reflect the connection, thank you for pointing it out.

2.21. The mean ISI score at baseline was not that elevated. The impact of this on results obtained should be discussed.

*Response*: noted, considered in the discussion, even though ~70% of participants were above the clinical cutoff with a different degree of severity of symptoms.

2.22. In Table 3, several mean scores fall in the “mediocre” range. Yet, the authors conclude that “most of the participants were very satisfied with the treatment and the app. I think it is important to tone down a bit that kind of interpretation.

*Response*: thank you, noted that as well.

2.23. Please clarify this phrase: “…a discouraging level structure suggesting continuous sleep improvement” (p. 12, 1st paragraph).

*Response:* adjusted, thank you.

2.24. Please use Cohen’s criteria for characterizing the effect sizes obtained: small (d=0.2), medium (d=0.5) and large (d=0.8). As such, effect sizes are not in the “medium to high” range but medium at best. Please change the findings interpretation accordingly.

*Response:* adjusted, thank you for pointing it out.

2.25. It would be important to note that the PSQI score was still in the clinical range at T2.

*Response:* noted, thank you.

2.26. Please specify that the difference obtained on objective SOL was significant and that no other significant effect was found on objective sleep measures.

*Response:* adjusted, thank you.

Discussion

2.27. Sometimes the authors describe their intervention as being a “training”. I would rather use the terms “intervention” or “treatment”.

*Response:* adjusted, thank you.

2.28. Given the small effect obtained on fear of sleep, should the intervention target this aspect more specifically? How so?

*Response:* thank you for pointing it out. Indeed, it could, but fear of sleep was not the main target variable, and it showed overall low prevalence within the population, thus, the issue might be not as critical in comparison to other variables and maybe should not be separately targeted. Nevertheless, corresponding adjustments have been made in the text.

2.29. The authors emphasize the importance of targeting PTSD-specific content in the program, yet they did not mention whether or not that was included in their program.

*Response:* the program did not include specifically PTSD-targeted content, since we aimed at sleep disturbances. Corresponding adjustments have been made in the text.

2.30. One study limitation that is discussed is the possibility that participants who self-subscribed might have had more severe insomnia resulting in a selection bias. Personally, I don’t find this is a problem. As mentioned earlier, participants had on average low levels of insomnia and sleep disturbances as assessed with the ISI and the PSQI. Hence, this does not appear to be true. Besides, this is what we want when we develop an intervention, to attract patients who are the most likely to benefit from it, in other words, those who have more severe problems.

*Response:* One study limitation that is discussed is the possibility that participants who self-subscribed might have had more severe insomnia, resulting in a selection bias. Personally, I don’t find this is a problem. As mentioned earlier, participants had on average low levels of insomnia and sleep disturbances as assessed with the ISI and the PSQI. Hence, this does not appear to be true. Besides, this is what we want when we develop an intervention, to attract patients who are the most likely to benefit from it, in other words, those who have more severe problems.

2.31. What appears most important to emphasize to me in terms of selection bias is the fact that an app may have appealed more to younger individuals, i.e., those who are more at ease with technology, thus limiting the generalization of findings to older individuals.

*Response:* thank you for pointing it out, corresponding adjustments have been made.
