# Supplementary material for: Non-guided, mobile, CBT-I-based sleep intervention in War-torn Ukraine: A feasibility study
Source: PLoS One. 2025 May 27;20(5):e0310070. doi: 10.1371/journal.pone.0310070 (PMC12111256; doi:10.1371/journal.pone.0310070)
Supplement: Attachment — Submitted filename: Response_to_Reviewers_auresp_2.docx [file pone.0310070.s005.docx]

Response to Reviewers:

Dear Reviewers. We highly appreciate your detailed feedback on the revised version of the manuscript. In addition to the revision of the mentioned aspects (see below), we have also done general improvements throughout in consistency of term use and writing. Corresponding revised sections are also provided here in the response for the ease of navigation.

Reviewer 1:

Introduction

1.1 A better expression for “low threshold”, that does not need to be defined, is “low intensity” intervention.

*Response*: Thank you for the comment, we have replaced the term *"low threshold"* with *"low intensity"* in the Introduction to enhance clarity and avoid the need for additional definitions.

*As indicated above, one of the major advantages of mobile-based CBT interventions in Ukraine is their low intensity, meaning they require minimal effort or resources from users, such as the need for travel, specialized equipment, or significant time commitments.*

1. 2. High uptake, feasibility and acceptance were hypothesized but are still not operationalized. The authors mention that these don’t need to be defined in pilot studies, a statement with which I disagree. If no criteria is stated a priori, then it is hard to conclude whether the intervention is or is not feasible and well accepted.

*Response:* We acknowledge this important point. To address this, we have explicitly operationalized uptake, feasibility, and acceptance by defining how these were measured in our study. These definitions have been added to the Introduction.

*Thus, we tested our preregistered hypotheses expecting high levels of engagement, feasibility, and acceptance. Uptake was defined as the proportion of registered users who completed the intervention. Feasibility was measured through participant engagement, including app usage and completion of required exercises and diary entries. Acceptance was evaluated based on participant satisfaction scores regarding the app's usability, relevance, and subjective effectiveness.*

1.3. In what language and for what kind of population was Sleep2 initially developed?

*Response:* Sleep2 was originally developed in German for a general adult population. This information has been added to the Introduction.

*The novel, evidence-based smartphone application Sleep2 (formerly called NUKKUAA®) comprises an unguided, fully automated CBT-I-based sleep treatment program. Sleep2 was initially developed in German for a general adult population but was later adapted for Ukrainian users to ensure cultural and linguistic relevance.*

Methods

1.4. The description of the time points (i.e., study design) in the text is still unclear to me. Please rework that section for more clarity.

*Response:* We have restructured the explanation of the study design to improve clarity. The updated version now clearly outlines the time points and their respective roles in the study*.*

*This study was planned as a single-arm, open-label, uncontrolled pre-post evaluation study (see Fig 1). The study design consisted of three key assessment points: (1) baseline assessment (T0) conducted after one week of accommodation and adaptation, (2) mid-study engagement tracking (T1), which marked the beginning of the 6-week CBT-I intervention, and (3) post-intervention assessment (T2) conducted at the end of the program. Throughout the intervention, participants completed daily sleep diaries and, if available, recorded heart rate data.*

1.5. I don’t understand this : “…sleep stages were not reported due to their limited generalizability”. Please clarify.

*Response:* Thank you for pointing this out. The original phrasing did not fully explain the rationale behind excluding sleep stage reporting. We have now clarified that since sleep stages are individually defined for each individual, we could not compute an average or compare stages between participants in a meaningful way.

*Sleep stages were not reported because heart rate-based classification provides individualized sleep stage data that varies significantly between participants. Since there is no standardized way to aggregate or compare sleep stages across individuals without introducing inaccuracies, we focused on broader, validated sleep parameters such as sleep onset latency, number of awakenings, and total sleep time. Instead of reporting aggregated sleep stage data, participants received personalized feedback on their sleep patterns based on their individual nightly variations.*

Results

1.6. In Table 4, several mean scores fall in the “mediocre” range, including “Improved sleep”. Still, the authors conclude that “most of the participants were very satisfied with the treatment and the app”. I think it would be important to point out the aspects on which the participants were less satisfied. The conclusion should also be adjusted accordingly to this somewhat low subjective global impression of sleep improvements.

*Response:* We have revised the Results section to acknowledge the aspects where participants were less satisfied (e.g., chatbot responses, push notifications, and perceived sleep improvements). However, we did not include the details into the conclusion in the abstract due to space limitations*.*

Participants were generally satisfied with the treatment and the app, particularly with content clarity, relevance, relaxation exercises, and program structure. However, several aspects received lower ratings, such as chatbot responses, push notifications, and perceived sleep improvement. These results indicate that while the program was well-received overall, certain features require refinement to enhance user experience and engagement.

1.7. Please identify in the text on which instruments the post-treatment values were still in the clinical range.

*Response:* We agree that it is important to specify which symptoms remained clinically relevant post-treatment. We have added this clarification in the Results section.

Completers reported statistically significant improvements on all symptoms after the sleep intervention, including sleep disturbance, insomnia, fear of sleep, anxiety, depression, PTSD, perceived stress, and somatic symptoms, with medium effect sizes (Table 5, see also density distributions and clinical cutoffs in S1 Fig). However, post-treatment values for insomnia, PTSD, and depression remained above clinical cutoffs for a substantial proportion of participants, indicating that while improvements were evident, some individuals continued to experience clinically relevant symptoms.

Discussion

1.8. In the study limitations, when discussing the other factors that could explain improvements over time, which could not be controlled for, given the lack of a control group, I would add the effect of treatment expectancies.

*Response:* Thank you, this has been added (page 14)

1.9. The presence of clinically meaningful sleep problems was not required for participation. Also, the authors recognize that insomnia and sleep difficulties were not severe at baseline. In my first review, I asked the authors to take that into account when interpreting the data. Indeed, one may not expect the same magnitude of effect sizes if a significant proportion of participants were non-clinical cases at baseline (and if insomnia scores were mild). On this, the authors replied that it probably did not make any difference. Yet, effect sizes obtained appear smaller to me than what is generally found in CBT-I trials. What would be the other reasons for that? For me, the most likely explanation remains the low insomnia severity at baseline but if the authors still disagree then I would expect them to identify other reasons.

*Response:* Thank you for your attention to this important point: we now highlight that lower baseline insomnia severity, the unguided nature of the intervention, and the burden of war may have contributed to reduced effect sizes.

Program completers reported substantial reductions in subjective sleep disturbance (minus 22.60%), insomnia severity (minus 35.08%), and fear of sleep (minus 32.43), all with low to medium-large effect sizes. There were also notable decreases in anxiety (minus 27.72%), depression (minus 28.67%), PTSD (minus 32.41%), somatic symptoms (minus 24.52%), and perceived stress (minus 17.90%). However, compared to traditional face-to-face CBT-I interventions, the effect sizes in our study appear somewhat smaller (42). This may be due to several factors, including the lower baseline severity of insomnia in our sample, the unguided nature of the intervention, and the absence of personalized therapist support. Additionally, the high psychological burden of ongoing war conditions may have limited participants’ ability to fully engage with and benefit from the intervention, thus reducing its overall effectiveness. Future studies should explore ways to enhance treatment engagement, such as incorporating tailored motivational support or hybrid approaches combining digital and human guidance.

1.10. Please tone down the conclusion (last paragraph) and emphasize the preliminary nature of the findings (as in the abstract).

*Response:* Thank you, we have modified both the first paragraph of the Discussion and the final paragraph to ensure that the findings are framed as preliminary due to the study’s uncontrolled design

Despite these limitations, scalable mobile-based interventions remain crucial for the Ukrainian population, particularly in war-threatened areas with internet and electricity access. Our study suggests that such interventions can be delivered and accepted, but findings are preliminary due to the study design. Further research, including randomized controlled trials, is needed to establish the efficacy of mobile-based CBT-I in Ukraine

Reviewer 2:

2.1. The Authors have addressed nearly all question raised. I believe that one issue, however, still needs to be further addressed. Again I specify that the psqi does not define sleep disorders; even if the authors have elaborated on this issue, it is incorrect to speak of scores >5 as sleep disorders; I believe that it should be interpreted as sleep difficulties, at most.
Furthermore it should be specified on what bases sample is divided in sleep disturbances and chronic sleep problems; I suppose that sleep disturbante is PSQI >5, buy chronic sleep problems?

*Response:* Thank you for pointing this out. Please, let us elaborate more on the matter:

First, we do not interpret an elevated PSQI score as defining sleep disorders in any section of the manuscript.

Second, we have specified how we operationalize the PSQI score and it is the following:

*Subjectively perceived sleep quality was assessed using the 19-item Ukrainian version of the PSQI (28). A global score greater than 5 was used as the cut-off to indicate poor sleep quality. This measure was operationalized as sleep disturbance because the PSQI’s global score is widely interpreted as reflecting clinically significant sleep problems, encompassing factors such as sleep continuity, latency, and disruptions. Referring to these issues as "sleep disturbance" aligns with the interpretation of a global score >5 as indicative of sleep-related difficulties that affect overall sleep quality.*

Third, we have adjusted the differentiation in the Table 3 and indicated only the cutoff of 5 (as instructed in the scale itself) to differentiate between individuals with normal sleep quality and those experiencing sleep disturbances, without categorizing them as having a sleep disorder.

We hope our explanation in this case is justified.
