# Supplementary material for: Non-guided, mobile, CBT-I-based sleep intervention in War-torn Ukraine: A feasibility study
Source: PLoS One. 2025 May 27;20(5):e0310070. doi: 10.1371/journal.pone.0310070 (PMC12111256; doi:10.1371/journal.pone.0310070)
Supplement: Attachment — Submitted filename: Response_to_Reviewers_auresp_3.docx [file pone.0310070.s006.docx]

Response to Reviewers:

Dear Reviewers. We highly appreciate your detailed feedback on the revised version of the manuscript. Please see our responses below. For your convenience, significantly-changed parts have been also provided in the responses.

Reviewer 1:

1.1. Regarding the “sleep disturbance” issues, now it is clearer. However, I suggest replacing “sleep disturbances” with “sleep problems” or “sleep difficulties”, throughout the text. With this terms there is no confusion or ambiguity with “sleep disorders”.

*Response*: We highly appreciate clarification regarding what causes the confusion. We decided to use sleep problems as suggested throughout the manuscript.

1.2. Line 34: I suggest removing “at least”. The impact of stressful situations and stressors in general imply- besides the presence of objective event- a subjective evaluation of the experience, that is less “tremendous scale” occurrence could also result in stress response involving sleep and health issues.

*Response*: Noted, replaced with especially, we believe it specifies the variability of the potential ‘scales’ of the conflict.

1.3. Line 43: the full stop at the end of sentence is missing.

*Response*: Appreciate the detail, added.

1.4. Line 88: please insert reference.

*Response*: Corresponding reference has been added.

1.5. Line 92: maybe “low intensity” is not the right term, please change it and explain better.

*Response*: We have replaced with minimal logistical and time demands, which should be clear. More detailed explanation has been retained, hope it is clearer now.

As indicated above, one of the major advantages of mobile-based CBT interventions in Ukraine is their minimal logistical and time demands, as they do not require travel, specialized equipment, or intensive time commitment – features that are critical in war settings where resources and mobility are usually limited.

1.6. Line 167: Fig. 1 description should be below the figure, not above.

*Response*: Adjusted, thank you.

1.7. Line 315: I suggest inserting data of t0 also in the text, besides the tables, regarding the baseline symptoms.

*Response*: Thank you, details have been added.

At t0, completers reported high levels of sleep problems (M = 8.93, SD = 3.45), low to medium levels of insomnia (M = 11.98, SD = 5.65), anxiety (M = 7.36, SD = 3.81), depression (M = 11.82, SD = 5.48), PTSD symptoms (M = 27.27, SD = 15.21), perceived stress (M = 7.38, SD = 3.18), and somatic symptoms (M = 9.42, SD = 4.73), as well as low levels of fear of sleep (M = 5.55, SD = 6.67; see Table 3).

1.8. Line 356: specify also in the text the values of improvements of symptoms.

*Response*: Thank you, values have been added.

Completers reported statistically significant improvements on all symptoms after the sleep intervention. Specifically, sleep problems decreased by 22.60% (from M = 8.93 to M = 6.91), insomnia by 35.08% (from M = 11.98 to M = 7.78), fear of sleep by 32.43% (from M = 5.55 to M = 3.75), anxiety by 27.72% (from M = 7.36 to M = 5.34), depression by 28.67% (from M = 11.82 to M = 8.43), PTSD symptoms by 32.41% (from M = 27.27 to M = 18.42), somatic symptoms by 24.52% (from M = 9.42 to M = 7.10), and perceived stress by 17.90% (from M = 7.38 to M = 6.06). Effect sizes ranged from small to medium (see Table 5 and S1 Fig).

1.9. Line 362: indicate the percentual of subjects that remained at clinical cutoff levels.

*Response*: Thank you, these details have been added.

However, post-treatment values for insomnia, PTSD, and depression remained above clinical cutoffs for a substantial proportion of participants: specifically, 41.82% still reported clinically significant insomnia (ISI > 7), 32.42% remained above the PTSD threshold (PCL-5 > 31), and 39.39% continued to show depressive symptoms above the clinical cutoff (PHQ-9 > 5). This indicates that while improvements were evident, a notable portion of participants continued to experience clinically relevant symptoms.

1.10. Line 393: please report the values of subjects that experienced discomfort in sensor use.

*Response*: Corresponding values have been added.

As for qualitative feedback, 38% of sensor users (25 out of 65) reported that the sensor restricted sleeping positions, was uncomfortable to adjust and wear, or caused some degree of discomfort. Additionally, some participants mentioned occasional synchronization and battery issues.

1.11. Line 401: it is not correct to say that “veridical feedback on objective sleep parameter can be very helpful for insomnia” since insomnia is a subjective disorder and does not require polysomnography for evaluation or treatment; it is recommended only in case is suspected other sleep disorder in differential diagnosis.

*Response*: Added more details, the adjusted text is listed below and highlighted in the revised manuscript for your convenience.

Although objective feedback on sleep parameters is not required for diagnosing or treating insomnia, some participants reported it as helpful in raising awareness and motivating behavioral change. Nevertheless, insomnia remains a subjective condition, and objective assessments such as heart rate-based sleep tracking are generally recommended only when other sleep disorders are suspected. Importantly, the delivery and daily use of HR sensors pose a logistical hurdle that could limit scalability.

1.12. Line 419-421: are not available nap info from the diaries?

*Response*: Unfortunately, yes, the data about naps could not be retrieved and was not available. We provided as clear clarification as possible: ‘However, this is a technical limitation as the algorithm has not been trained on nap data and, therefore, these activities could not have been included into the program, as well as into the analysis.’

1.13. Line 435: also this 9% of subjects completed the program?

*Response*: Yes, we have provided more details in the text.

Regarding the pathology of the sample and whether we addressed the program’s target group: our sample demonstrated significant sleep impairments, confirming that we reached the intended population – only 9% of the completers (14 out of 160) did not report sleep problems at baseline, meaning that even these individuals chose to complete the full program, potentially seeking general sleep improvement or stress reduction.

1.14. Line 437: specify the “elevated levels” values of sleep difficulties (i.e., the PSQI score), also for chronic sleep problems.

*Response*: Thank you for pointing it out, we have extended this part.

Before starting the intervention, participants who completed the program exhibited significantly elevated levels of sleep problems. Specifically, 60% of completers scored above the clinical cutoff on the PSQI (score >5; M = 8.93, SD = 3.45), indicating clinically relevant sleep difficulties. Moreover, 30% had PSQI scores greater than 10, which is often interpreted as indicating more severe or chronic sleep problems. Regarding insomnia symptoms, 44% of participants met criteria for subthreshold insomnia and 27% for clinical insomnia based on the ISI, with scores above 7 (M = 11.98, SD = 5.65).

1.15. 455: please specify the different level of insomnia with the study referenced (42).

*Response*: Thank you, corresponding values have been added.

1.16. Line 465-467: please delve a little deeper by explaining the subjective nature of insomnia disorder.

*Response*: Hope we have understood your comment correctly and provided corresponding details.

The striking discrepancy between subjective and objective sleep measures is a well-documented phenomenon, particularly in insomnia, which is fundamentally a subjective disorder. Insomnia is defined by a person’s self-reported difficulties falling or staying asleep and the associated daytime impairment, regardless of whether objective measurements (e.g., actigraphy or polysomnography) confirm these problems. This often leads to a mismatch between perceived and physiologically measured sleep – a pattern referred to as sleep-wake state misperception or subjective-objective sleep discrepancy.

Reviewer 2:

Thank you for taking into consideration our revision efforts.
